# Culturally Tailored Cognitive Training for Older Chinese Americans: Preliminary Results from a Pilot RCT

**DOI:** 10.1093/geroni/igaf122.4281

**Published:** 2025-12-31

**Authors:** Tingzhong (Michelle) Xue, Aybey Amy Wei, Camilla Sanders, Runqi Wangqin, Bei Wu, Eleanor McConnell, Hanzhang Xu

**Affiliations:** University of Massachusetts Amherst, Amherst, Massachusetts, United States; Duke University, Durham, North Carolina, United States; Duke University, Durham, North Carolina, United States; Duke University, Durham, North Carolina, United States; NYU Shanghai, Shanghai, China (People’s Republic); Duke University, Durham, North Carolina, United States; Duke University, Durham, North Carolina, United States

## Abstract

Older Chinese Americans are at increased risk of developing dementia but have limited access to culturally relevant prevention programs due to linguistic and transportation barriers. To address these issues, we have worked with older Chinese Americans and their adult children to co-design a mobile-based cognitive training intervention. In this study, we evaluated the feasibility and acceptability of this cognitive training intervention through a pilot randomized controlled trial with baseline, 8-, and 12-week follow-ups. Older Chinese Americans aged 60+ with consent capacity were randomized into the intervention (n = 20) or the wait-list control group (n = 10). The Intervention group completes 10 - 15 minutes training daily for 12 weeks from 21 training exercises covering cognitive domains including attention, processing speed, memory and visual spatial ability etc. Feasibility and acceptability were measured by retention rate and training completion rate, as well as a satisfaction survey. We enrolled our participants from January-July 2025. Of 30 participants (mean age 71.2±8.9 years; 60% female), 46.6% had less than college education and 96.7% had limited English proficiency. To date, 22 out of 30 participants have completed the study. The retention rate was 100% in the intervention group and 70% in the control group. Participants in the intervention group rated satisfaction as 9.7 out of 10. We are scheduled to complete all follow-ups and secondary outcomes, including cognition and psychological well-being by mid Oct 2025. Our preliminary evidence suggests that this culturally and linguistically tailored cognitive training intervention is feasible and acceptable among older Chinese Americans.

